# Petroleum in Scabies

**Published:** 1865-04

**Authors:** 


Petroleum in Scabies.—M. Decaisne, of Antwerp, treats itch by simply spreading this oil all over the body of the patient. He has obtained excellent results, and finds that the emanations from the oil purify the clothes which are put on immediately after the operation. He dispenses with the soap, the bath, the sulphur, and the liquid sulphuret of lime used in the Belgian army.

